# Examining the effect of intermittent cycling throughout a 3-h period on peripheral blood concentrations of haemopoietic stem and progenitor cells and cytolytic natural killer cells

**DOI:** 10.1186/s13287-025-04261-1

**Published:** 2025-03-28

**Authors:** Phoebe A. Cox, Fendi Pradana, Ella Noble, Samuel J. E. Lucas, Guy Pratt, Mark T. Drayson, Kevin Amin, Francesca A. M. Kinsella, Alex J. Wadley

**Affiliations:** 1 School of Sport, Exercise, and Rehabilitation Sciences, University of Birmingham, Birmingham, UK; 2 Institute of Immunology and Immunotherapy, University of Birmingham, Birmingham, UK; 3 Birmingham Centre for Cellular Therapy and Transplantation, University Hospitals Birmingham NHS Foundation Trust, Birmingham, UK; 4 Clinical Immunology Service, University of Birmingham, Birmingham, UK; 5 Nutrition Study Program, Tadulako University, Palu, Indonesia

**Keywords:** HSPC, Peripheral blood stem cell donation, Exercise, Interval cycling, Natural killer cell, Transplantation, Feasibility

## Abstract

**Background:**

Peripheral blood stem cell (PBSC) donation is the primary procedure used to collect haemopoietic stem and progenitor cells (HSPCs) for haemopoietic stem cell transplants (HSCT), however there is a clinical need to reduce collection times and achieve sufficient HSPC doses for successful engraftment. Short bouts of interval cycling transiently enrich peripheral blood with HSPCs and cytolytic natural killer (CD56^dim^ NK) cells, which predict engraftment success and prevent post-transplant complications respectively. Despite this, feasible protocols for use during PBSC collections (≈ 3 h) have yet to be evaluated.

**Methods:**

In a randomised crossover design, 18 adults (9 young: 22.7 ± 3.2 years, 9 older: 65.2 ± 12.9 years) completed 3 × 3-h trials: high-intensity interval exercise (HIIE, 9 × 2-min cycling at 80–85% heart rate (HR)max/9 × 18 min rest), moderate-intensity interval exercise (MIIE, 9 × 4-min cycling at 65–70% HRmax/9 × 16 min rest) and REST (180 min). Immune cell subsets, including HSPCs and CD56^dim^ NK concentrations (cells/µL) were determined across 18 timepoints and area under the curve (AUC, cells/µL x minutes) and total cell dose (cells/kg) were estimated.

**Results:**

By design, MIIE elicited lower average and peak HR and rating of perceived exertion than HIIE and was reported as more enjoyable. All cell subset concentrations increased following each interval of MIIE and HIIE. Across all participants, the estimated cell dose of total lymphocytes, monocytes, T cells, CD56^bright^ and CD56^dim^ NK was greater in MIIE and HIIE versus REST (*p* < 0.03), but there were no differences between MIIE and HIIE. The magnitude of change versus REST was greatest for CD56^dim^ NK versus all cell subsets, and AUC was significantly greater in HIIE versus REST for this cell type only (*p* < 0.0001). There were no statistically significant differences in HSPC AUC (*p* = 0.77) or cell dose (*p* = 0.0732) in MIIE and HIIE versus REST. Age did not predict any changes across trials or timepoints for any cell type.

**Conclusion:**

Persistent mobilisation of peripheral blood immune cells throughout 3 h of MIIE and HIIE evoked sustained numbers of CD56^dim^ NK cells, but there was no reliable difference in HSPCs compared to a time-matched period of rest.

**Supplementary Information:**

The online version contains supplementary material available at 10.1186/s13287-025-04261-1.

## Introduction

Over 90,000 haemopoietic stem cell transplants (HSCT) are performed worldwide each year and have become the primary treatment option for people with haematological malignancies [1]. The self-renewal and regeneration capacity of haemopoietic stem and progenitor cells (HSPCs) ensures haemopoietic restoration following myeloablative conditioning regimens [2, 3]. The principal procedure used for collecting HSPCs is peripheral blood stem cell (PBSC) donation, and this is undertaken by either the patient (autologous), or a human leukocyte antigen (HLA) matched healthy donor (allogeneic) [4]. Prior to PBSC collection, all donors receive subcutaneous injections of Granulocyte Colony Stimulating Factor (G-CSF) for 4–5 days to stimulate proliferation of HSPCs in the bone marrow and subsequent mobilisation into peripheral blood. Sub-optimal HSPC doses are common after administering G-CSF in autologous donors (~ 40% achieve < 2 × 10^6^/kg) [5], resulting in poor HSPC engraftment and prolonged treatment regimens [6, 7]. Over 95% of allogeneic donations meet clinical threshold for HSPCs after G-CSF (> 4 × 10^6^/kg) [8], but the composition of the collected immune graft influences the risk of infection, disease relapse and Graft versus Host Disease (GvHD) [9, 10]. Notably, lower numbers of CD56^dim^ natural killer (NK) cells [11] monocytes [12] regulatory T cells [13] and gamma-delta T cells [14] have been reported as independent predictors of GvHD incidence. Despite the clinical success of Plerixafor infusions alongside G-CSF since 2008 [15], current clinical trials are examining the efficacy of novel chemokine receptor antagonists to improve PBSC collections (e.g., MGTA-145) [16]. This highlights an unmet clinical need to improve HSPC dose and immune composition during apheresis.

Single bouts of exercise rapidly mobilise HSPCs and other immune cells into peripheral blood [17, 18] and this has long garnered interest in whether exercise might complement PBSC collections to enhance HSPC dose and immune composition. However, exercise-induced mobilisation of immune cells such as HSPCs [19], monocytes [20], regulatory T cells [21] gamma-delta T cells [22] and CD56^dim^ NK cells [23] is transient, and for HSPCs the peak concentration achieved is significantly lower than following G-CSF treatment (~ 2 vs. ~ 30-fold) [24]. Moreover, apheresis sessions can take 3–4 h, limiting the utility of continuous exercise during PBSC donations. Recently, Pradana et al. [25] reported that just 2 × 2-min bouts of interval cycling at 95% of maximal heart rate (HR_max_), and 3 × 4-min bouts at 84% HR_max_ increased HSPC concentrations compared to 30 min of steady-state continuous cycling at 70% HR_max_. These responses were comparable to peak HSPC concentrations reported after continuous exercise [17, 26, 27], and the final interval of other high intensity interval exercise (HIIE) protocols [28, 29]. This study also reported that the homing propensity of HSPCs was largely preserved in peripheral blood after HIIE and accompanied by markedly higher numbers of CD56^dim^ NK cells. These data, and others [17, 26], indicate that exercise intensity is the principal mediator of the HSPC and cytotoxic effector cell responses to exercise. Immune composition can be altered after very short periods of cycling, but whether these intervals can be interspersed over a PBSC collection, which last ≈3 h, is unclear.

Despite the physiological potential of interval exercise to improve immune composition, high-intensity exercise may not be feasible for some of the general population serving as allogeneic donors, and autologous donors burdened with cancer related morbidity and therapy. Nevertheless, there are data to support the safety and tolerability of regular HIIE for people with cancer as part of prehabilitation for surgery, chemotherapy, radiotherapy [30], and more recently prior to allo-HSCT [31, 32]. Enrolled participants in these two studies were adherent to the prescribed HIIE sessions (both 92%), and gave positive feedback; however, Kuehl et al. [32] reported recruitment rates as ‘much too low’. Participants in this study were older (55 ± 11) and indeed auto-HSCT are predominantly used to treat cancers diagnosed in people over the 60 years of age, with patients experiencing fatigue, nausea and bone/joint pain [33, 34]. Moderate intensity interval exercise (MIIE: defined as 50–70% HR_max_) is an alternative to HIIE that has been reported as more tolerable than HIIE by older adults [35, 36] and would likely be more feasible during PBSC collections, particularly for autologous donors. Whilst previous studies have reported reduced HSPC mobilisation after exercise in older versus younger adults [37], others have reported no difference [38]. Most importantly, it is not yet clear whether HSPCs and other immune cell numbers can be sustained with HIIE or MIIE over 3 h.

Therefore, the primary aim of this study was to evaluate the impact of HIIE and MIIE interspersed throughout a 3-h period on changes in peripheral blood HSPC and CD56^dim^ NK cell concentrations in younger versus older adults, compared to a period of rest. Serial blood sampling was employed every 10 min across the 3 h to examine dynamic changes in peripheral blood cell concentrations, total area under the curve and estimated total cell dose expressed relative to body mass. The feasibility and acceptability of each cycling protocol were also examined. Our hypothesis was that HSPC, and CD56^dim^ NK cell concentrations would be greater throughout a 3-h period of HIIE versus MIIE, but that MIIE would be perceived as less difficult and more enjoyable than HIIE.

## Materials and methods

### Participants

Our preliminary data were used to calculate sample size based on changes in mean (and standard deviation (SD)) peripheral blood HSPC concentrations after 2 intervals of HIIE [25]. This study was powered a priori using GPower 3.1.9.7 to detect an effect size of *d* = 0.52, with an α of 5% and 80% power. Accordingly, eighteen participants provided written informed consent to participate in this study, including nine older adults (> 50 years, OA) and nine younger adults (18–30 years, YA). The exclusion criteria included smoking (including vaping), hypotension, body mass under 50 kg, body mass index (BMI) over 40 kg/m^2^ or history of cardiovascular, metabolic, respiratory, or neurological disease. Furthermore, only participants who had not donated blood in past 3 months and had not taken anti-inflammatory drugs and/or steroids within 14 days were included. The General Practice Physical Activity Questionnaire (GPPAQ) [39] was implemented to only include ‘moderately active’ or ‘inactive’ participants (defined by undertaking < 150 min of structured physical activity per week) and ability to partake safely in exercise was determined using the Physical Activity Readiness Questionnaire (PAR-Q) [40]. This study was given favorable ethical opinion by the Science, Technology, Engineering and Mathematics Ethical Review Committee at the University of Birmingham (ERN_19-1574PA7) and conformed to the Declaration of Helsinki, except for prior registration on a publicly accessible database.

#### Study design overview

**Fig. 1:**
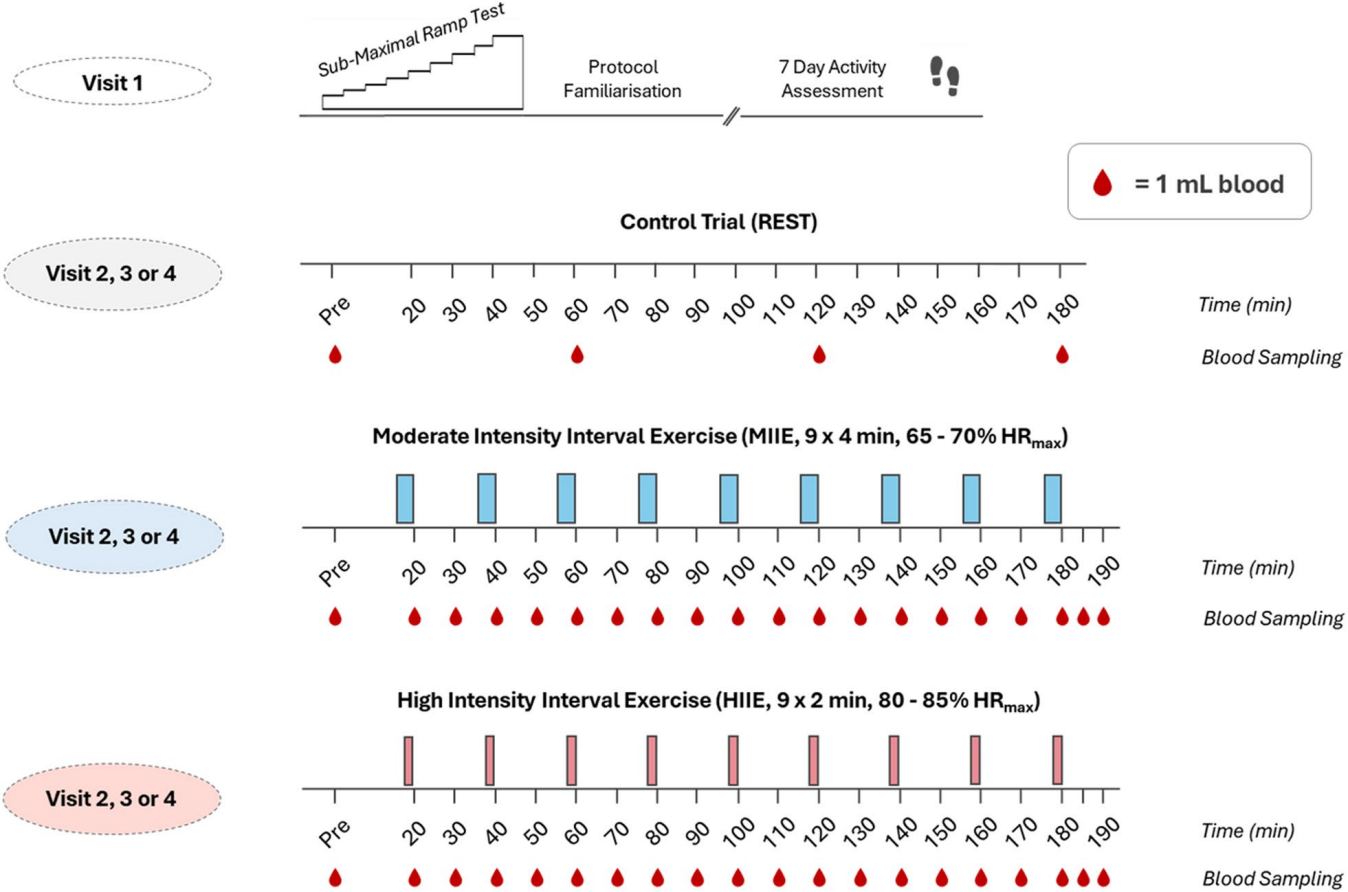
Study design overview. Including screening and blood sampling procedures of the three randomised trials: REST, control seated rest trial; MIIE, moderate intensity interval exercise trial; HIIE, high intensity interval exercise trial. Blood sampling is indicated with a blood droplet (1 mL) for whole blood analysis. Other participant procedures (e.g., fluid and food ingestion) are detailed in the methods section

The study was a randomised crossover design, consisting of four visits to the School of Sport, Exercise and Rehabilitation Sciences, University of Birmingham (Fig. 1). Both groups initially performed a submaximal exercise tolerance test (reaching 85% of age predicted HRmax; [41] on a 45° reclined cycle ergometer (Ergoselect 1200, Ergoline, Germany). For the OA group, an electrocardiogram (ECG) was recorded at rest and throughout exercise to identify any cardiac arrhythmias or abnormalities, determined by a qualified physician. Three randomised experimental timed trials were then performed on a 45° reclined cycle ergometer, comprising moderate-intensity interval exercise (MIIE: 9 × 4-min, 65–70% HRmax), high-intensity interval exercise (HIIE: 9 × 2-min, > 85% HRmax) and a REST trial (Rest: 45° supine position), each separated by seven days. To control for external lifestyle factors that may affect immunity, a seven-day recall of illness symptoms, sleep quality, fatigue, and anxiety were recorded on each visit.

### Preliminary measures

The OA group completed an initial screening visit consisting of a resting 12-lead ECG (Cardiosoft, Vyaire, USA). Prior to the submaximal exercise tolerance test, participants were asked to refrain from strenuous exercise for 48 h. Following a 15-min period of rest upon arrival, height, weight, heart rate (Polar Verity Sense, Polar, Finland) and blood pressure were measured. Participants undertook a 5-min warm up, with Watts increasing every minute to achieve a rating of perceived exertion (RPE) of 11 (fairly light) [42]. Following this, workload increased by 10 W every 2 min until participants reached 85% of their age predicted maximum heart rate (HRmax), calculated using 211- (0.64 × age) [43]. A 12-lead ECG was used in OA to monitor for irregular heart rhythms during exercise (Cardiosoft, Vyaire, USA). Workloads for subsequent experimental trials were then calculated using a linear regression between heart rate and workload. Following 15 min of rest, participants underwent a familiarisation session to confirm that the calculated power elicited a HR in the desired range for each trial. Each participant was then fitted with a tri-axial accelerometer (Geneactiv, Activinsights, UK) to wear for 7 days leading up to the first experimental trial.

### Experimental trials

For seven days prior to each experimental visit, participants completed a daily illness log to document any signs or symptoms of illness using a 7-point scale [44]. Participants were asked to refrain from vigorous exercise for 48 h and alcohol and caffeine for 24 h prior to all experimental trials. A 24-h food diary was completed before the first experimental visit and participants were asked to replicate this diet prior to each remaining trial. Experimental trials started between 08:00am-09:00am and were conducted under stable climatic conditions (temperature: 20.7 ± 0.6 °C, humidity: 29 ± 2%, barometric pressure:1005 ± 1.7 hPa) at least one week after the submaximal exercise tolerance test. All participants arrived following an overnight fast (from 9 pm the previous night) and were given a standardised breakfast, which consisted of porridge oats and semi-skimmed milk (normalised for carbohydrate content—0.5 g per kg of body mass). Following 15 min of rest, height, weight, blood pressure and a baseline blood sample were taken. Participants then completed the State Trait Anxiety Inventory (STAI) [45], Multidimensional Fatigue Inventory (MFI) [46] and Pittsburgh Sleep Quality Index (PSQI) [47] questionnaires. The STAI scale ranged from < 35 (low anxiety) to > 65 (high anxiety), MFI was composed of 5 subscales (general fatigue, physical fatigue, mental fatigue, reduced motivation, and reduced activity) that were totalled to determine fatigue between 20 and 100, with a higher score indicating more fatigue. Finally, PSQI was categorised into 7 components (sleep: quality, latency, duration, efficiency, disturbance; use of sleep medication and daytime dysfunction), and the sum of these gave a global PSQI score between 0 and 21, with a higher score indicating poorer sleep quality. Furthermore, a score of ≥ 80% indicates good sleep efficiency.

For each experimental trial, an intravenous cannula was placed into the antecubital vein of the arm. The cannula was flushed with 2 mL of isotonic saline (0.9% sodium chloride; PosiFlush, BD, United States) following each blood sample to prevent blood clotting in the cannula. For HIIE and MIIE, participants then completed a 5-min warm-up, with power progressively increased to elicit an RPE of 11. Following the warmup, each cycling interval was performed every 20 min over a 180-min period, totalling 9 bouts. Heart rate was monitored continuously throughout all experimental trials. HIIE intervals consisted of a one-minute build up phase (50% HRmax) and then participants cycled for 2-min at 80–85%HRmax. During MIIE, participants cycled for 4-min at 65–70% HRmax and REST consisted of participants lying in a 45-degree supine position for 180-min and undertaking no cycling intervals. In all trials, a small snack was provided at 100 min and a fixed volume of water (500 mL) for the entire trial, with consumption limited to every 60 min. During each trial, subjective exertion and the affective response were measured every 10 min after 20 min using the RPE and Feeling scales respectively [48].

### Protocol feasibility and acceptability

Adherence to each interval during MIIE and HIIE was defined as cycling continuously at the set power output (1 = adherent; 0 = not adherent) and total adherence expressed as a percentage of the completed nine intervals. Compliance was defined as completing intervals 4–9 at the prescribed % HRmax (1 = compliant; 0 = not compliant) and total compliance expressed as percentage of compliant versus non-compliant intervals (not reaching HR or missed interval). At the end of the study, participants completed an acceptability survey, self-reporting their levels of enjoyment and difficulty throughout MIIE and HIIE.

### Blood sampling

For HIIE and MIIE, blood samples were taken after a 15-min period of rest (Pre), the first interval (20 min) and then every 10 min thereafter (30, 40, 50, 60, 70, 80, 90, 100, 110, 120, 130, 140, 150, 160, 170 min), capturing responses immediately after each interval and after 10 min of passive recovery. A blood sample was taken after the final interval (Post) and then 5 and 10 min into recovery (Post + 5 and Post + 10). Blood samples were taken every hour during REST (Pre, 60, 120, and 180 min). At each timepoint, 1 mL of blood was collected into potassium ethylene diaminetetracectic acid (EDTA) vacutainer tubes. Before each blood sample, 2 mL of blood was drawn and discarded to ensure acquisition of peripheral blood in real-time and not an artifact of residual blood in the cannula. Whole blood lymphocyte, monocyte and haemoglobin counts were acquired using an automated haematology analyser (Yumizen H500, Horiba, Japan).

### Flow cytometry

HSPCs and CD56^dim^ NK cells in whole blood were identified using a Cytoflex-S flow cytometer (Beckman Coulter, California, USA). All antibodies were purchased from BioLegend (San Diego, CA) and data analysis performed using CytExpert v2.5 Software (Beckman Coulter, California, USA). Compensation was applied for each trial using compensation beads (UltraComp eBeads, ThermoFisher Scientific, Massachusetts, USA) and single stained controls. CD56^dim^ NK cell gates were created using fluorescence minus one (FMO) controls. Dead cells were excluded from analysis using 7-amino-actinmyosin D (7-AAD).

Enumeration of peripheral blood HSPC concentration was performed using a volumetric flow cytometry method validated by the International Society of Hematotherapy and Graft Engineering (ISHAGE) [49]. Anti-human CD34-PE (clone: 581), anti-human CD45-FITC (clone: 2D1) and 7-AAD were added to 100 µL of whole blood in the dark, at room temperature for 20 min and then 2 mL of red blood cell lysis buffer added for 10 min (BioLegend, San Diego, CA). All samples were then analysed within 1 h. A Boolean gating strategy (Supplemental Fig. 1) was used to enumerate HSPCs (CD34^+^ CD45^dim^ SSC^low^), with a minimum of 100 events acquired and concentration expressed as cells/µL.

To identify T and NK cell subsets, whole blood (100 µL) was stained with anti-human CD3-FITC (clone: HIT3a), anti-human CD56-PE (clone: 5.1H11), anti-human CD16-APC (clone: 3G8) and 7-AAD as above, but samples were then washed twice in FACS buffer (500 mL of D-PBS, 2 mM EDTA, 0.1% Sodium Azide and 1 mL FCS) for 5 min at 500×*g* and 21° C before sample acquisition. The gating strategy is outlined in Supplemental Fig. 2, with T cells identified as CD3^+^ and the CD3^−^ gate used to define regulatory (CD16^−^ CD56^bright^) and cytolytic (CD16^+^ CD56^dim^) NK cells, for which a minimum of 5,000 events were acquired for the latter. The peripheral blood concentration (cells/µL) of each subset was then calculated by coupling the cell frequency determined by flow cytometry with whole blood lymphocyte count.

### Data and statistical analysis

GraphPad Prism 10.2.2 analysis software (San Diego, CA) was used to perform statistical analysis and to create figures. Residuals for the outcomes were explored using histograms and all data examined for normal distribution using the Shapiro–Wilk test. Data were either pooled (N = 18) or stratified by age (YA, N = 9 vs. OA, N = 9) for analysis. Cell concentrations were analysed as an average or peak or over time (0–180 min) and by trial (REST, MIIE and HIIE) and age (younger vs. older) using mixed-effects analysis of variance. Due to the high volume of pairwise comparisons, only Time x Trial interaction, trial effects or significant comparisons between pre-exercise and post-interval timepoints were included. Area under the curve (AUC, cells/µL x minutes) for each cell type was calculated using the trapezoidal method, whereby the change in area between every timepoint was totalled for each 180-min trial [50]. An estimated total cell dose was calculated by multiplying the average peripheral blood cell concentration (cells/ µL) from the 18 sampling timepoints by an estimated total blood volume (TBV, µL) and expressed per kg of body mass (cells/kg) [51]. TBV was derived from the Nadler equation, with specific equations employed based on biological sex [52]. For this calculation, body mass and height were fixed based on measurements at rest, but haemoglobin concentrations at each timepoint were examined to account for relative shifts in blood volume during each trial, based on an adaption of the Dill and Costill equation (51). Recovery time points (Post + 5 and Post + 10) in MIIE and HIIE were not included in AUC, total cell dose or average concentration analysis.

Habitual physical activity level data were extracted (GENEActiv Software, Activinsights, UK) and analysed (R and RStudio, Posit, PBC, Boston, Massachusetts) using specialist software and Hildebrand activity cut-off points [53]. Only participants recording a minimum wear time of 16 h per day for 4 weekdays and 1 weekend day were included in data analysis. Post hoc analyses of any interaction effects were performed by a test of multiple comparisons, using Sidak or Tukey correction. A one-way analysis of variance (One-way ANOVA) was used to calculate effect sizes. All values are presented as means ± standard deviation (SD). Statistical significance was accepted at the *p* < 0.05 level. Effect sizes (Cohen's *d*) were calculated for primary outcomes (HSPC and CD56^dim^ NK cells) by dividing the difference between means by the pooled standard deviations and 95% confidence intervals. Effect sizes of 0.2, 0.5 and 0.8 were considered small, moderate and large respectively [54, 55].

## Results

### Group characteristics

Participant characteristics stratified by age are displayed in Table 1, with a mean difference of 42 ± 13 years between OA and YA (*F*_(8, 8)_ = 15.92, *p* < 0.0001). There were no significant differences in body mass, height, BMI, blood pressure, daily steps, light physical activity and sedentary time, between OA and YA (*p* > 0.05), but MVPA was higher in YA vs. OA (P = 0.0003).

**Table 1:** **|** Participant characteristics

Variable	Older adults (N = 9)	Younger adults (N = 9)	*p* value
Age (years)	65 ± 13	23 ± 3*	< 0.0001
Body mass (kg)	75.4 ± 14.3	75.0 ± 14.8	0.953
Height (cm)	165.1 ± 11.6	172.4 ± 7.5	0.132
BMI (kg. m^2^)	27.5 ± 3.7	25.1 ± 4.0	0.551
Systolic BP (mmHg)	131 ± 22	126 ± 13	0.618
Diastolic BP (mmHg)	78 ± 9	74 ± 9	0.431
Daily Steps	8308 ± 3276	11,184 ± 2233	0.054
Daily MVPA (hours)	1.80 ± 0.57	3.19 ± 0.67*	0.0003
Daily light PA (hours)	3.81 ± 1.41	3.54 ± 1.23	0.690
Daily sedentary (hours)	10.78 ± 1.95	9.20 ± 1.95	0.114

Data displayed as mean ± SD

*Significant difference between YA and OA (*p* < 0.05)

BMI, body mass index; BP, blood pressure; MVPA, moderate to vigorous physical activity; PA, physical activity

Changes in participant lifestyle habits between experimental visits were examined using repeated measures ANOVAs. There were no significant differences in illness symptoms (*F*_(2, 51)_ = 1.000), *p* = 0.375), fatigue (*F*_(1.834, 31.18)_ = 2.809, *p* = 0.080), sleep efficiency (*F*_(1.775, 30.17)_ = 0.317, *p* = 0.705) state (*F*_(1.641, 27.90)_ = 0.2056, *p* = 0.772) or trait (*F*_(1.998, 33.96)_ = 0.244, *p* = 0.784) anxiety levels between REST, MIIE and HIIE. Therefore, these variables were not used as co-variates in subsequent cell analyses.

### Physiological responses during trials

**Fig. 2:**
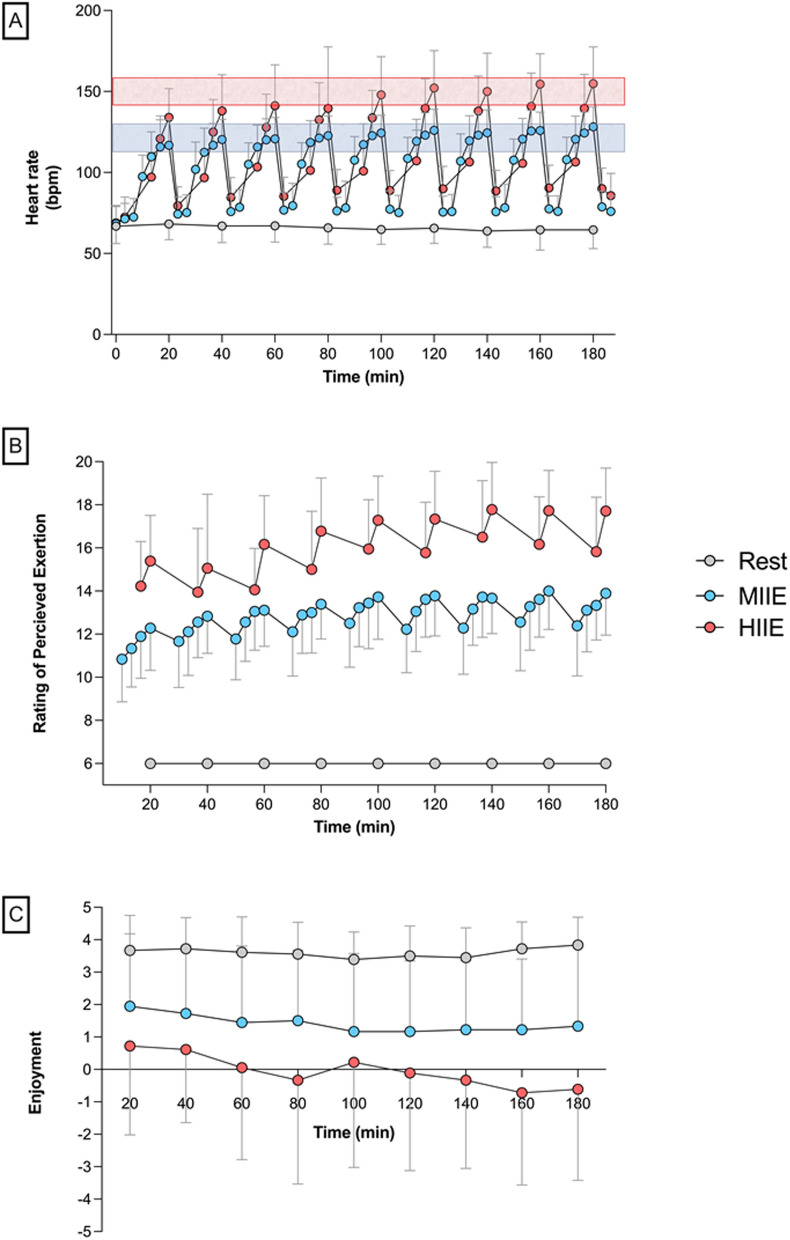
Heart rate (**A**) rating of perceived exertion (**B**) and the affective response (**C**) across the three trials in all participants pooled (N = 18): REST (grey circles), MIIE (blue circles) and HIIE (red circles). Red and blue shaded boxes represent the mean HR region for HIIE (80– 85% HR_max_) and MIIE (65–70% HR_max_) across all participants. Values are mean ± SD. Pairwise comparisons are not included

On average, participants cycled within the prescribed HR zones at 85.0 ± 7.5% HR_max_ and 67.7 ± 3.6% HR_max_ for HIIE and MIIE respectively. Across all intervals, peak HR (Time x Trial Interaction: *F*_(16, 407)_ = 10.28, *p* < 0.0001) and RPE (Time x Trial Interaction: (*F*_(8, 271)_ = 2.451, *p* = 0.014) were greater throughout HIIE > MIIE > REST (*p* < 0.0001; Fig. 2). Adherence to MIIE and HIIE (i.e., completion of intervals at prescribed power) was 100% and 99.4% respectively. Furthermore, participants were 95% compliant (i.e., cycling within target HR zone for intervals 4–9) during MIIE and 84% during HIIE. Total estimated energy expended (kcal) during the trials was similar between MIIE and HIIE; however, a two-way ANOVA (*F*_(1, 16)_ = 7.765, *p* = 0.013) revealed that estimated energy expenditure was significantly greater in YA than OA during MIIE (*p* = 0.011), and within YA, MIIE was greater than HIIE (*p* = 0.034).

### Changes in peripheral blood cell concentrations

A mixed effects model revealed no significant differences in haemoglobin concentrations between MIIE and HIIE versus REST and therefore subsequent cell concentration data were not adjusted for changes in blood volume (Time x Trial Interaction: *F*_(38, 958)_ = 1.155, *p* = 0.242). There were largely no differences between YA and OA across the dataset. Therefore, pooled data are presented and age effects highlighted where relevant.

#### HSPCs

**Fig. 3:**
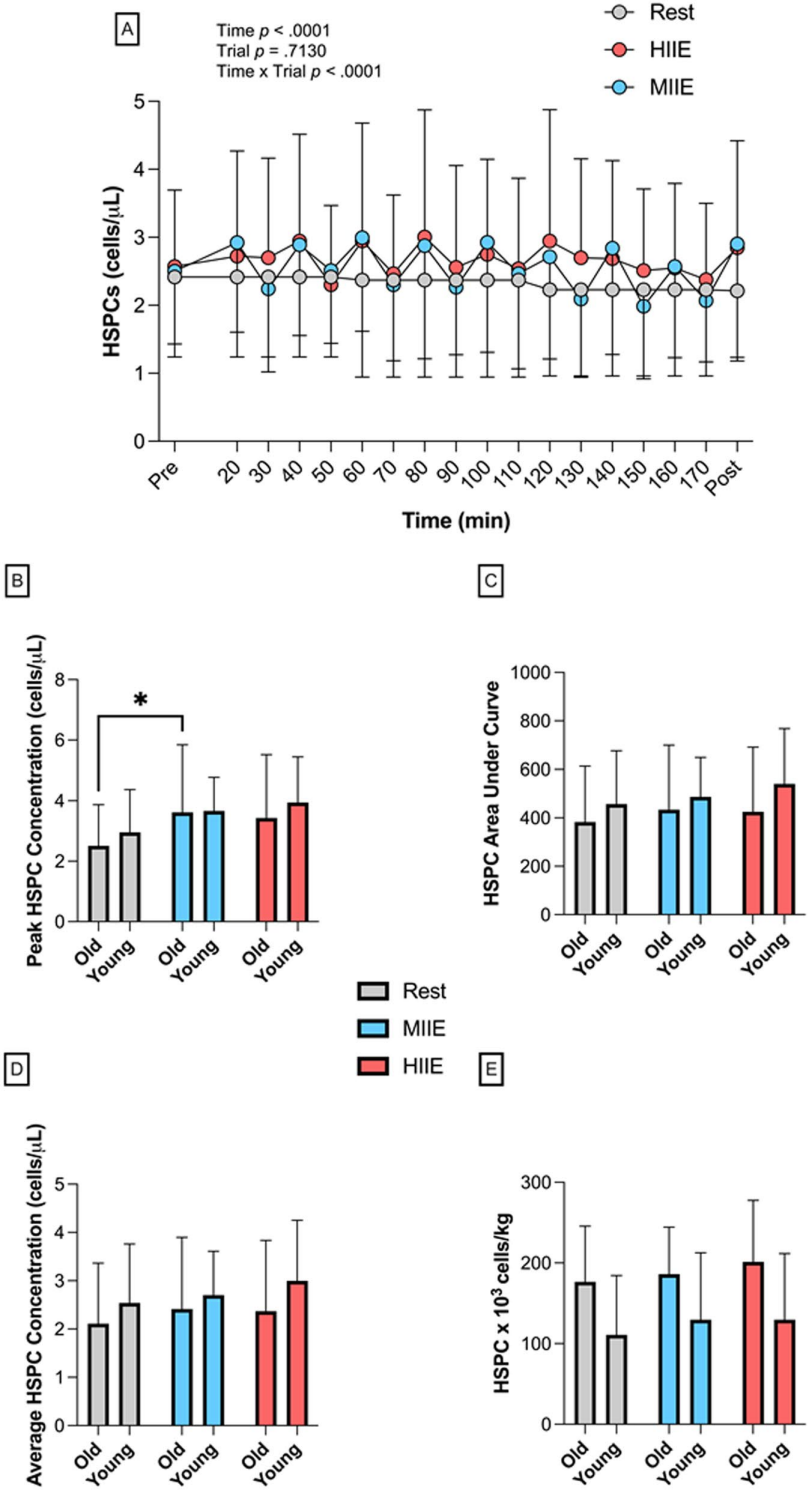
Peripheral blood HSPC concentrations (cells/µL) across 18 timepoints in all participants pooled (N = 18) (**A**) and then stratified by age (old, N = 9 vs. young, N = 9) for peak HSPC concentrations (**B**) total area under the curve (cells/µL x minutes) (**C**) average HSPC concentration (**D**) and HSPC dose (cells per kilogram of body mass) (**E**) in REST (grey circles and bars), MIIE (blue circles and bars) and HIIE (red circles and bars). Values are mean ± SD. * indicates significant differences between trials: *p < 0.05. Pairwise comparisons are not included on panel A

Peripheral blood HSPC concentrations over time during REST, MIIE and HIIE are shown in Fig. 3. A one-way repeated measures ANOVA revealed a significantly higher peak (*F*_(1.638, 27.84)_ = 11.13, *p* = 0.006) and average post-interval (*F*_(1.871, 31.81)_ = 5.513, *p* = 0.01) peripheral blood HSPC concentration in MIIE (peak: *p* = 0.003, *d* = 0.6; post-interval: *p* = 0.018, *d* = 0.4) and HIIE (peak: *p* = 0.008, *d* = 0.6; post-interval: *p* = 0.05, *d* = 0.4) vs. REST. A Time x Trial interaction effect was observed (*F*_(34, 860)_ = 2.421, *p* < 0.0001), with post hoc analysis revealing significant changes during MIIE (notably post-exercise timepoints 40, 60, 140 and 160 min, *p* < 0.03) and HIIE, but not REST. Following MIIE and HIIE, HSPC concentrations at Post + 5 and Post + 10 were not significantly different to Pre (*p* > 0.05) Total AUC was then calculated to reflect fluctuations in HSPC concentrations over each 180-min trial (cells/µL x minutes). There was a mean difference in AUC for HIIE (471.1 ± 235.6) > MIIE (460.5 ± 214.9) > REST (419.8 ± 221.6), but there were no significant differences between trials (*F*_(2, 51)_ = 0.3526, *p* = 0.705; MIIE vs. REST *d* = 0.2; HIIE vs REST *d* = 0.3) (Fig. 3). A similar pattern was observed for estimated total HSPC dose adjusted for body mass (cells per kg: MIIE, 157.9 ± 75.42, *d* = 0.2 and HIIE 165.5 ± 85.23, *d* = 0.3 vs. REST, 143.7 ± 77.08), although these differences were not statistically significant (*F*_(1.924, 32.71)_ = 2.826, *p* = 0.073). When stratifying by age, peak HSPCs were greater in MIIE versus REST for OA only (*p* = 0.047), although there were no differences across trials between YA and OA.

#### * CD56*^*dim*^* NK cells *

**Fig. 4:**
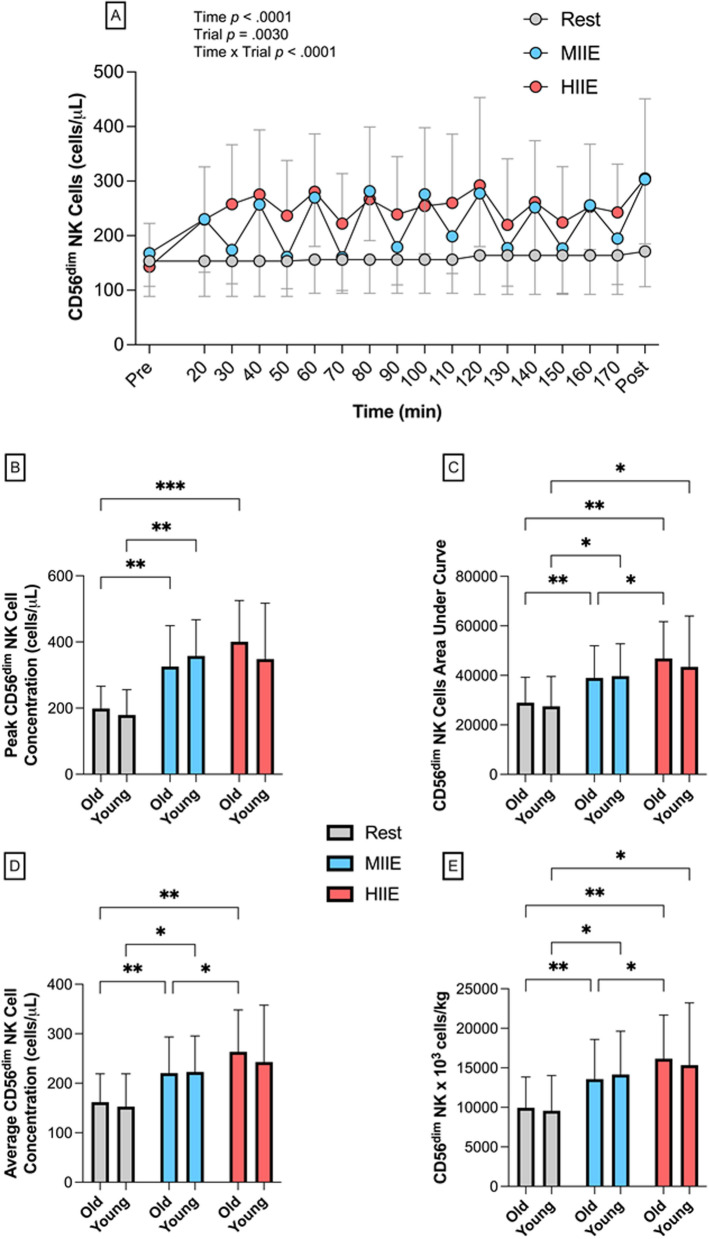
Peripheral blood CD56^dim^ NK cell concentrations (cells/µL) across 18 timepoints in all participants pooled (N = 18) (**A**) and then stratified by age (old, N = 9 vs. young, N = 9) for peak CD56^dim^ NK cell concentrations (**B**) total area under the curve (cells/µL x minutes) (**C**) average CD56^dim^ NK cell concentration (**D**) and CD56^dim^ NK dose (cells per kilogram of body mass) (**E**) in REST (grey circles and bars), MIIE (blue circles and bars) and HIIE (red circles and bars). Values are mean ± SD. * indicates significant differences between trials: *p < 0.05. # indicates a significant difference between old MIIE vs old HIIE, *p* < 0.05. Pairwise comparisons are not included on panel A

Peripheral blood CD56^dim^ NK cell concentrations across REST, MIIE and HIIE are shown in Fig. 4. A one-way repeated measures ANOVA revealed a significantly higher peak (*F*_(1.619, 27.52)_ = 18.00, *p* < 0.0001) and average post-interval (*F*_(1.360, 23.12)_ = 11.22, *p* = 0.001) peripheral blood CD56^dim^ NK cell concentration in MIIE (peak: *p* < 0.0001, *d* = 1.3; post-interval: *p* < 0.0001, *d* = 1.5) and HIIE (peak: *p* < 0.0001, *d* = 1.4; post-interval: *p* < 0.0001, *d* = 1.3) compared to REST. A Time x Trial interaction effect was observed (*F*_(34, 765)_ = 5.331, *p* < 0.0001), with post hoc comparisons revealing no changes in REST and significantly higher CD56^dim^ NK cell concentrations within MIIE at 60, 80, 100, 120, 140, 160 180 min versus Pre (*p* < 0.02) and HIIE at 20, 30, 40, 60, 90, 110, 120, 140, 160, 170, 180 min versus Pre (*p* < 0.028). CD56^dim^ NK cell concentrations were significantly greater in HIIE versus REST at post-exercise and between interval timepoints after 10 min of passive rest (20, 30, 40, 50, 60, 80, 90, 110, 120, 140, 160, 170, 180 min, *p* < 0.045). There were significantly greater CD56^dim^ NK cells at post-exercise timepoints only in MIIE versus REST (20, 40, 60, 80, 100, 120, 140, 160, 180 min, *p* < 0.03). Differences between HIIE and MIIE were minimal, with only two timepoints greater in HIIE versus MIIE (30 min and 50 min, *p* = 0.040). Following MIIE, CD56^dim^ NK cell concentrations at Post + 5 and Post + 10 were not significantly different to Pre (*p* > 0.05). After HIIE, the concentration of CD56^dim^ NK cells at Post + 5 were sustained above Pre (*p* = 0.0016), but not at Post + 10 (*p* = 0.10).

A one-way ANOVA revealed significantly (*F*_(2, 48)_ = 6.592, *p* = 0.003) greater AUC for HIIE versus REST (cells/µL x minutes: 45,239 ± 17,259 vs. 28,268 ± 10,839, *p* = 0.002, *d* = 1.2) but not MIIE versus REST (39,318 ± 12,595, *p* = 0.07, *d* = 0.9), and average CD56^dim^ NK cell concentration (*F*_(1.277, 21.71)_ = 8.134, *p* = 0.006) was higher in HIIE (6.52 ± 3.86, *p* < 0.0001, *d* = 1.2) and MIIE (6.14 ± 3.76, *p* < 0.0001, *d* = 1.0) versus REST (4.89 ± 3.04). Similarly, when estimating total CD56^dim^ NK cell dose for body mass (cells/kg), MIIE (13,492 ± 4192, *p* < 0.0001, *d* = 1.03) and HIIE (15,449 ± 5780, *p* < 0.0001, *d* = 1.24) were significantly higher than REST (9566 ± 3409, *F*_(1.841, 29.46)_ = 28.03, *p* < 0.0001). When stratifying by age, average (*p* = 0.033), AUC (*p* = 0.039) and estimated dose (*p* = 0.031) of CD56^dim^ NK cells were significantly greater in HIIE versus MIIE for OA only (Fig. 4), but there were no differences across trials between YA and OA.

#### Lymphocytes and monocytes

**Fig. 5:**
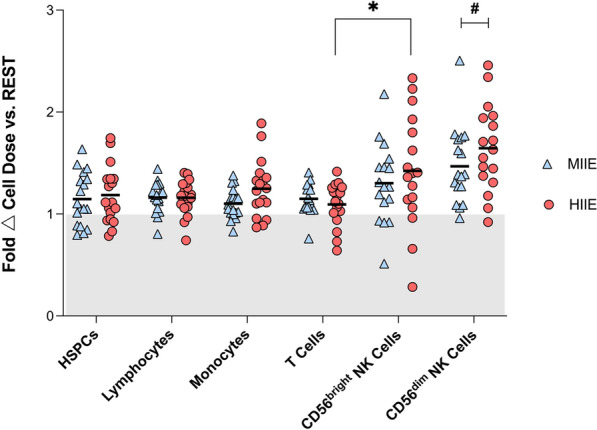
Fold change in estimated total dose of immune cell subsets in MIIE and HIIE vs REST in N = 18. Values are mean ± SD. * indicates significant differences between subsets: * *p* < 0.05. # indicates a significant difference between CD56^dim^ NK cell dose vs HSPCs, lymphocytes, monocytes and T cells in HIIE and MIIE

Peripheral blood concentrations of total lymphocytes, CD56^bright^ NK cells, T cells and monocytes were calculated, with average and peak values, AUC and estimated cell dose (cells/kg) reported in Table 2. Peak concentrations of all cell types were significantly higher in MIIE and HIIE versus REST and the same pattern occurred for average cell concentrations of lymphocytes and CD56^bright^ NK cells. The estimated cell dose of lymphocytes and monocytes was significantly greater in MIIE (*p* < 0.04) and HIIE (*p* < 0.01) versus REST, whilst the dose of CD56^bright^ NK cells was higher in HIIE versus REST (*p* = 0.05), and for T cells MIIE versus REST (*p* = 0.02). The magnitude of change in CD56^dim^ NK cell dose in MIIE and HIIE versus REST was significantly greater than all subsets except CD56^bright^ NK cells (Fig. 5). Despite greater AUC for lymphocytes, monocytes, CD56^bright^ NK, and T cells in response to MIIE and HIIE compared to REST, these changes were not significant. There were no differences across trials between YA and OA.

**Table 2:** Concentrations and estimated total trial responses for lymphocytes, monocytes, regulatory natural killer and T cells

		Trial			
Cell subset		REST	MIIE	HIIE	*p* value
Lymphocytes	Average (cells/μL)	1554 ± 307^a,b^	1796 ± 413	1796 ± 423	0.0002
	Peak (cells/μL)	1720 ± 379^a,b^	2284 ± 553	2211 ± 556	< 0.0001
	AUC	279,700 ± 55,323	321,567 ± 73,619	321,131 ± 74,482	0.12
	Cells (10^3^)/kg	94,916 ± 17882^a,b^	109,532 ± 23,462	109,740 ± 24,918	0.0002
Monocytes	Average (cells/μL)	454 ± 127^b^	491 ± 116	561 ± 194	0.01
	Peak (cells/μL)	521 ± 149^a,b^	641 ± 155	712 ± 252	0.001
	AUC	81,453 ± 22,635	87,736 ± 20,799	100,632 ± 34,782	0.10
	Cells (10^3^)/kg	27,757 ± 7684^a,b^	29,923 ± 6815	34,101 ± 10,864	0.0071
CD56^bright^ NK	Average (cells/μL)	4.9 ± 3.1^a,b^	6.1 ± 3.8	6.5 ± 3.9	0.01
	Peak (cells/μL)	5.9 ± 3.2^a,b^	8.9 ± 5.6	9.3 ± 5.3	0.0003
	AUC	901 ± 541	1100 ± 670	1177 ± 699	0.44
	Cells (10^3^)/kg	302 ± 193^b^	378 ± 234	400 ± 239	0.0121
T Cells	Average (cells/μL)	1024 ± 232^a^	1,173 ± 318	1,110 ± 311	0.02
	Peak (cells/μL)	1163 ± 280^a,b^	1531 ± 465	1434 ± 433	< 0.0001
	AUC	184,607 ± 41,699	209,520 ± 56,695	198,283 ± 55,125	0.38
	Cells (10^3^)/kg	62,366 ± 13362^a^	71,304 ± 18,383	67,705 ± 18,757	0.0253

Data displayed as mean ± SD

^a^Significant difference between REST and MIIE (*p* < 0.05)

^b^Significant difference between REST and HIIE (*p* < 0.05)

Abbreviations: NK, natural killer; AUC, area under curve

### Subjective protocol feasibility perceptions

There was a significant difference in the affective response between the three trials (Time x Trial Interaction: *F*_(16, 408)_ = 1.964, *p* = 0.014). For every interval, participants perceived MIIE (*p* < 0.019) and HIIE (*p* < 0.019) as less enjoyable than REST, with HIIE consistently having the lowest affective rating, but this was not statistically lower than MIIE (*p* > 0.07). There were no differences in the affective response between OA and YA.

Upon finishing the study, participants completed an acceptability survey. HIIE was rated significantly more difficult than MIIE (*p* < 0.0001), with 56% of participants preferring MIIE over HIIE. There were no differences between OA and YA (*p* > 0.26). Participants experienced greater symptoms of thirst during HIIE than MIIE (*p* = 0.007), however hunger did not differ between HIIE and MIIE (*p* = 0.10).

## Discussion

Findings from the present study indicate that throughout a 3-h period, cycling intervals of high (HIIE) and moderate intensity (MIIE) evoked repeated mobilisation of HSPC and CD56^dim^ NK cells, but the sustainability of these responses differed compared to a time-matched period of rest. Estimations of total cell dose across the 3-h periods (cells/kg) indicated significantly higher total lymphocytes, monocytes, T cells, CD56^bright^ and CD56^dim^ NK cells in HIIE and MIIE versus REST, and although this pattern was observed for HSPCs, the differences were not statistically significant (*p* = 0.07). By design, HIIE was more physiologically demanding than MIIE, as reflected by HR and RPE data and self-reported as more difficult and less enjoyable. Nonetheless, adherence and compliance throughout HIIE and MIIE was high and particularly, all cellular, physiological and subjective responses did not differ between older and younger adults. Collectively, these data demonstrate that repeated modulation to immune composition induced by 18–36 min of cycling intervals interspersed throughout a 3-h period led to sustained numbers of peripheral blood mononuclear cells, but not reliably for HSPCs.

It has been speculated that acute modulation of immune composition during bouts of exercise offers potential as a clinical adjuvant in the treatment of some cancers, and harvesting HSPCs during PBSC collections is one such application. However, practical steps to feasibly implement this have yet to be implemented. To the authors knowledge, this was the first study to examine the impact of interval cycling interspersed across a 3-h period on the sustainability of immune cell numbers in peripheral blood, principally HSPCs and CD56^dim^ NK cells. By prescribing brief cycling intervals based on clinical HIIE HR zones [36, 56] and substantial rest periods (16 and 18 min), these protocols were designed to elicit a physiological stimulus that was potentially manageable for both allogeneic and autologous donors, albeit in the absence of G-CSF.

Following intervals of MIIE and HIIE, average HSPC concentrations increased to 2.8 ± 1.4 and 2.9 ± 1.6 cells/µL respectively, which falls in the range of 2–6 cells/µL reported after bouts of HIIE [25, 28, 29] and continuous exercise [57, 58]. In the context of harvesting HSPCs, these ‘spikes’ in HSPC concentration represent only ‘snapshots’ of 9 timepoints over 3 h and importantly when concentrations were greatest versus pre-exercise. By enumerating HSPC concentrations between each interval, estimations of total AUC across 18 timepoints revealed no significant differences for HIIE and MIIE compared to REST. Furthermore, there were no significant differences in estimated total HSPC dose relative to body weight between trials, despite 13 of 18 participants exhibiting a higher HSPC dose versus REST. Further, while not significantly different, the mean dose was 9 ± 2% and 15 ± 11% higher in MIIE and HIIE versus REST respectively, however, the effect size was small-to-moderate (*d* = 0.3). Of note, participants undertook the trials without prior G-CSF infusion and thus estimated HSPC doses were approximately 10–20-fold lower than thresholds used clinically to predict successful HSPC engraftment [5, 8]. This lower dose was expected, as this study was designed as a proof of concept to initially establish whether HSPC concentrations could be sustained with intermittent cycling over 3 h. Nevertheless, despite repeated HSPC mobilisation after 9 bouts of short cycling intervals over 3 h, there was no significantly reliable difference in the total HSPC dose versus a time-matched period of rest.

Clinically, PBSC collection success is solely determined by HSPC dose; however, composition of other immune cells in the graft influences clinical endpoints after HSCT. For example, CD56^dim^ NK cell number has been associated with reduced risk of disease relapse [59] and GvHD incidence [11] after alloHSCT and superior overall survival and progression-free survival following autoHSCT in people with haematological malignancies [60]. Lymphocyte infusions are also commonly deployed after alloHSCT as an adoptive immunotherapy to prevent relapse [61, 62] and more recently NK cell specific infusions have been used therapeutically after autoHSCT (63, 64). In the present study, MIIE and HIIE evoked repeated mobilisation of total lymphocytes, T cells, CD56^dim^, CD56^bright^ and monocytes and this translated to greater total cell doses over 3 h during MIIE and HIIE versus REST (Table 2). CD56^dim^ NK cells exhibited the greatest difference across all cell types (Fig. 5), typical of a cytotoxic dependent mobilisation pattern, which is principally driven by greater cell surface expression of β2-adrenergic receptor [64, 65]. As NK cells are the first lymphocyte subset to recover after HSCT, increased graft numbers after cycling may impact upon engraftment and immune reconstitution [66]. Indeed, Maggs et al. reported that T cell depleted patients undergoing matched unrelated and sibling donor alloHSCT had a 90% lower 2-year relapse rate when the graft was enriched with a NK cell dose of > 6 × 10^6^ cells/kg [59]. This was primarily driven by the CD56^dim^ population and strikingly in the present study, CD56^dim^ NK dose was + 3.9 and + 5.9 × 10^6^ cells/kg higher in MIIE and HIIE versus REST respectively, thus indicating clinical significance. Furthermore, accumulating evidence indicates that exercise-mobilised NK cells exhibit a more cytotoxic and anti-tumor phenotype compared to cells isolated at rest [67]. Therefore, cycling during PBSC collections may both increase NK cell number, and augment a graft versus tumour effect [68].

It is noteworthy that the T cell dose was also significantly greater in MIIE versus REST (+ 8.9 × 10^6^ cells/kg), thus raising questions regarding alloreactivity and GvHD risk. Whereas NK cells exert their effector functions independent of major histocompatibility complex (MHC) or antibodies, T cells are MHC restricted [69]. Higher T cell numbers in the collected graft could promote histocompatibility differences with host T cells, and alloreactivity exaggerated by a higher proportion of antigen experienced T cells evoked by exercise, which is a reproducible finding [70, 71]. Collectively, these data demonstrate widespread changes to immune composition in response to 3 h of interval cycling, which could influence the early phases of immune reconstitution after transplantation [72].

Although the clinical significance of the cellular changes is presently unclear, a secondary aim of the present study was to examine the feasibility of performing these protocols over 3 h. As expected, HIIE evoked significantly greater physiological responses than MIIE (Fig. 2), and was reported as more difficult, less enjoyable and participants declared a greater degree of thirst. Despite HIIE being more challenging, adherence and HR compliance were not significantly different between MIIE and HIIE or when stratified by age and aligned with rates reported in studies utilising HIIE training for patients undergoing HSCT [31, 32]. It is noteworthy that pooled data (N = 18) demonstrated no differences in cell responses between MIIE and HIIE, and therefore substantially longer rest periods (16–18 min) between intervals over 3 h appeared to overcome the intensity-dependent mobilisation of HSPCs observed in previous studies [17, 25]. However, when stratified by age, average concentration, AUC and estimated dose of CD56^dim^ NK cells were greater in HIIE versus MIIE for OA only, and peak HSPC concentration was greater in MIIE versus REST for OA. There were no statistical differences between OA and YA, but these data may indicate greater sensitivity to NK cell and HSPC mobilisation in OA.

Mobius Winkler et al. previously examined changes in peripheral blood HSPC concentrations across 16 timepoints of a 4-h bout of continuous cycling at 70% anaerobic threshold [57]. HSPC concentrations incrementally increased throughout the bout, peaking 2.5-fold higher than before exercise, and were raised for the duration of the bout. Given that peak fold changes in HSPCs were 1.33 and 1.35 for MIIE and HIIE respectively in the present study, estimated cell dose was undoubtedly higher in the former study (estimated at + 114%), although the physiological demand and energy cost was substantially greater (estimated at 1753 vs. 189 kcal). MIIE and HIIE were adopted to provide a feasible protocol for participants, with application within a 3-h PBSC donation in mind, thus 16–18 min of rest were prescribed between cycling intervals. Given the cumulative effect of HIIE on HSPC concentration seen in our previous research [25] we hypothesized that intermittent cycling would sustain HSPC concentration across a 3-h period. However, the egress of lymphoid and myeloid cells occurs in as little as 2 min of exercise cessation [73], with HSPCs specifically returning to pre-exercise concentrations within 5–10 min [17, 58]. There is therefore a trade-off between the physiological potential of cycling to sustain elevated HSPC concentrations [57] and undertaking a protocol that is manageable within a 3-h window, particularly for autologous donors. Future studies should focus on refining the protocol design to minimise the rapid egress of HSPCs, but this would require prescribing shorter rest periods.

## Limitations/considerations

This study was not without limitations. All cell doses were estimated from a mean of 18 peripheral blood cell concentrations and TBV [52]. Cell doses were expressed relative to body mass, for which we applied only a single measurement at rest but adjusted for relative shifts in blood volume throughout the trials [51]. It is notable that our analysis only included snapshots of 18 timepoints over the trials, and this is not totally representative of an apheresis session whereby all cells are harvested after processing the donors blood volume 3–6 times over ≈3 h [74]. A 3-h protocol was chosen based on typical PBSC collection times; however, we acknowledge that the procedure may exceed 3 h, or require multiple days. For our analysis, we focused on enumerating HSPCs, monocytes and lymphoid subsets within peripheral blood, but other subsets, notably gamma delta T cells, regulatory T cells and cytotoxic T cells have been associated with post-transplant health outcomes [13, 14, 75] and warrant measurement in future studies. Furthermore, functional analysis of exercise-mobilised cells may offer insight into engraftment potential and clinical outcomes.

## Conclusions

The present study found that 4-min bouts of MIIE interspersed with 16-min rest periods, and 2-min bouts of HIIE interspersed with 18-min rest periods, throughout a 3-h period, induced repeated mobilisation of immune cells into the circulation. By estimating total cell dose, CD56^dim^ and CD56^bright^ NK cells, lymphocytes, T cells and monocytes were sustained above a period of rest in MIIE and HIIE for 3 h, however there was no reliable effect for HSPCs. The greatest relative change in cell dose was observed for CD56^dim^ NK cells and cell responses were largely not impacted by age. Marked changes to immune composition induced by intermittent cycling for 3 h may have important implications for health outcomes following HSCT. Therefore, these data provide a rationale to investigate the effects of cycling during PBSC donations with prior G-CSF administration.

## Supplementary Information

Supplementary Material 1.

## Data Availability

All data generated or analysed during this study are included in this published article and the supplementary materials.

## Abbreviations

HSPC: Hematopoietic stem and progenitor cell
PBSC: Peripheral blood stem cell
HSCT: Hematopoietic stem cell transplant
NK: Natural killer
OA: Older adults
YA: Younger adults
G-CSF: Granulocyte colony stimulating factor
MIIE: Moderate intensity interval exercise
HIIE: High intensity interval exercise
HR: Heart rate
RPE: Rating of perceived exertion
TBV: Total blood volume
AUC: Area under curve
GvHD: Graft versus host disease
alloHSCT: Allogeneic hematopoietic stem cell transplant
autoHSCT: Autologous hematopoietic stem cell transplant
HR_max_: Maximum heart rate
MHC: Major histocompatibility complex
