# Supplementary material for: Examining the effect of intermittent cycling throughout a 3-h period on peripheral blood concentrations of haemopoietic stem and progenitor cells and cytolytic natural killer cells
Source: Stem Cell Res Ther. 2025 Mar 28;16:155. doi: 10.1186/s13287-025-04261-1 (PMC11951530; doi:10.1186/s13287-025-04261-1)
Supplement: Supplementary file 1 — Supplementary Material 1. [file 13287_2025_4261_MOESM1_ESM.docx]

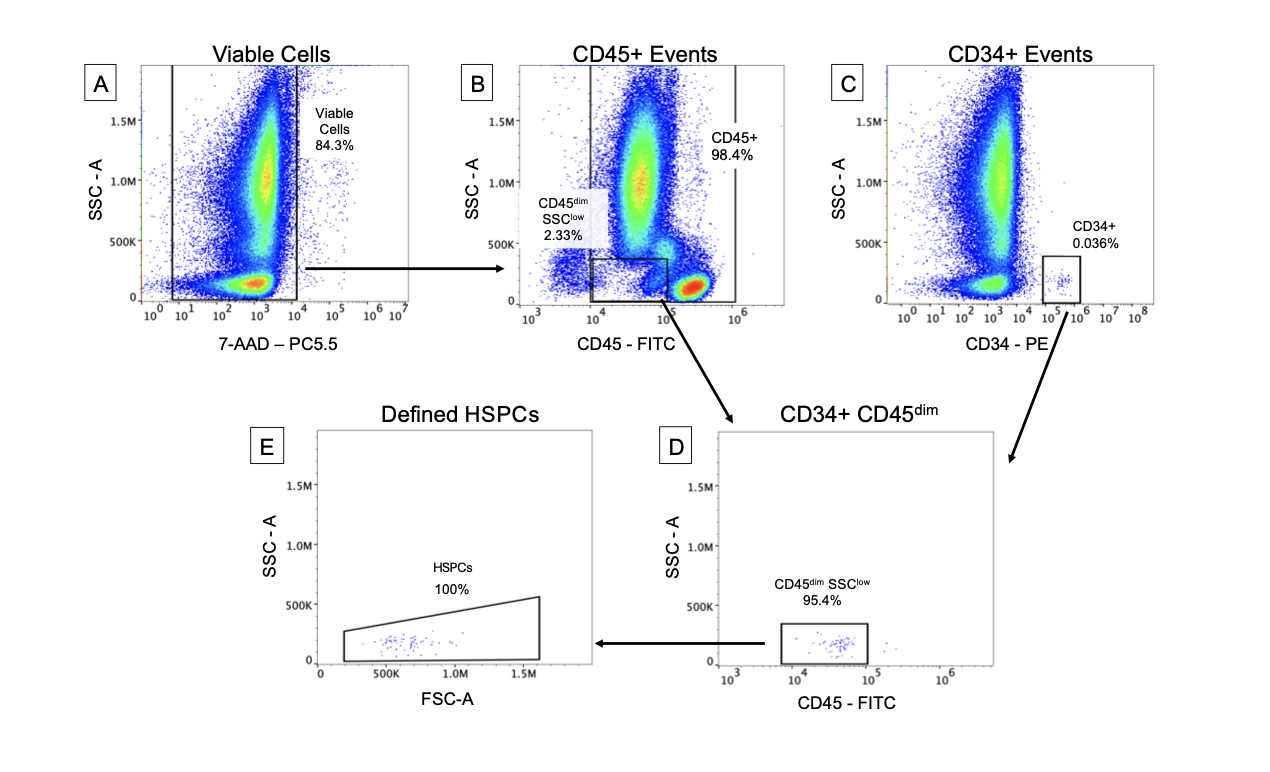
**Supplementary Figures 1 and 2**

**Supplementary Figure 1** | A Boolean gating strategy was used to enumerate HSPCs following the International Society of Hematotherapy and Graft Engineering (ISHAGE). From whole blood, CD34+ cells were identified by gating of viable cells using 7-AAD (A), CD45+ events (B) and then CD34+ events (C). From plot B, CD45 events with dim expression and low SSC were combined with CD34+ events (D). Viable HSPCs were defined as CD34^+^CD45^dim^SSC^low^ on a FSC vs. SSC plot (E).


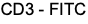

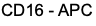


**Supplementary Figure 2** | This gating strategy was used to enumerate CD3+ T cells, CD56^dim^ and CD56^bright^ NK cells. From whole blood, CD3+ T cells (D) were identified by gating of singlets (A), lymphocytes using FSC vs SSC (B), viable cells using 7-AAD (C). Using plot C, CD3- events (D) were used to identify CD56^dim^ and CD56^bright^ NK cells by CD16+CD56^dim^ and CD16-CD56^bright^, respectively (E).
